# Complete mitochondrial genome and phylogenetic analysis of a *Chorthippus fallax* (Zuboxsky) isolated in rangeland of northwest region of China

**DOI:** 10.1080/23802359.2019.1695549

**Published:** 2019-12-09

**Authors:** Jialu Deng, Ziqi Yu, Jie Ning, Heng Wang, Xinda Lin, Xiaoli Liu

**Affiliations:** College of Life Science, Huaibei Normal University, Huaibei, China

**Keywords:** *Chorthippus fallax* (Zuboxsky), Mitogenome, Phylogeny

## Abstract

In this study, we assembled a complete 16,143 bp mitochondrial genome for *Chorthippus fallax* (Zuboxsky), which encodes 13 protein-coding genes, 22 tRNAs, 12S and 16S rRNAs and a 722 bp D-loop in the characteristic arrangement of superfamily Acrididae. The most common start codon for 13 PCGs is ATG and the most common termination codon is TAA. The overall A + T content was 75.13%. Phylogenetic analysis based on the entire mitochondrial genome indicated *C. fallax* (Zuboxsky) had more close relationship with *Euchorthippus fusigeniculatus* and *Chorthippus chinensis* in family Arcypteridae, and implied non-monophyly for the family. The data will be important for better understanding of the phylogenetic relationship of members in Acrididae superfamily.

Mitochondrial DNA (mtDNA) has many characteristics for using in phylogenetic analysis, such as maternal inheritance, relatively high evolutionary rate and conserved gene components (Li et al. 2019), and has been widely used. The typical insect mitogenome is a circular molecule of 14–19 kb in length and contains 13 protein-coding genes (PCGs), 22 transfer RNA (tRNA), two ribosomal RNA (rRNA) and a non-coding region (Wolstenholme 1992).

*Chorthippus fallax* (Zuboxsky) is a genus of Acrididae and broadly distributed in pasture areas Northwest China (Sun et al. 2015), causing large losses caused severe damage to rangelands and influenced the local economy of these regions (Sun et al. 2016). While *C. fallax* (Zuboxsky) is one of the most important grasshoppers and the limited molecular data restricted further progressing in this insect. The entire mitogenome of *C. fallax* (Zuboxsky) is not available and only sequences for cytb, COII and COI genes are deposited in GenBank so far.

In this study, we sequenced, assembled and annotated the mitochondrial genome for a *C. fallax* (Zuboxsky), which was collected on the alpine steppe (Altitude, 2846 m a.s.l.; 38° 02 N, 41 101° 34 E) of Sunan County, Gansu Province, China, marked as GS-18-5 and stored in Zoological Specimen Museum of Huaibei Normal University. Primers were designed by alignment of the mitogenomic sequences of other reported species. PCGs were determined by BLAST in NCBI and tRNA genes were identified using tRNAscan-TE search server (Schattner et al. 2005). The complete mitochondrial genome of *C. fallax* (Zuboxsky) is 16,143 bp in length (GenBank accession MK693137) and the gene organization and components are the same as other reported grasshoppers (Zhang et al. 2013). Like most other invertebrates, most genes of mitogenome of *C. fallax* (Zuboxsky) located at J-strand except 8 tRNA genes, 4 PCGs and rRNA genes. The lrRNA and srRNA genes consist of 1314 and 847 bp, respectively. The tRNA gene is from 64 to 71 bp. The most common start codon for PCGs was ATG and the most stop codon was TAA except ND1 and ND4 genes. The base composition, relative synonymous codon usage (RSCU) and nucleotide substitution statistics were analyzed using MEAG 7 (Kumar et al. 2016) and the results reflected A/T bias of the mitogenome as reported in other species (Kim et al. 2018). The mitochondrial base composition is A 42.86%, T 32.27%, C 14.28% and G 10.59%.

The phylogenetic position of *C. fallax* (Zuboxsky) was investigated using the entire mitogenomic sequences by the Maximum Likelihood method in MEGA 7.0 (Kumar et al. 2016) and the result was shown in Figure 1. Our phylogenetic analysis confirmed that *C. fallax* (Zuboxsky) belonged to family Arcypteridae and had a closer relationship with *Euchorthippus fusigeniculatus* and *Chorthippus chinensis*. Our results also implied non-monophyly for the Acrididae family.

**Figure 1.**
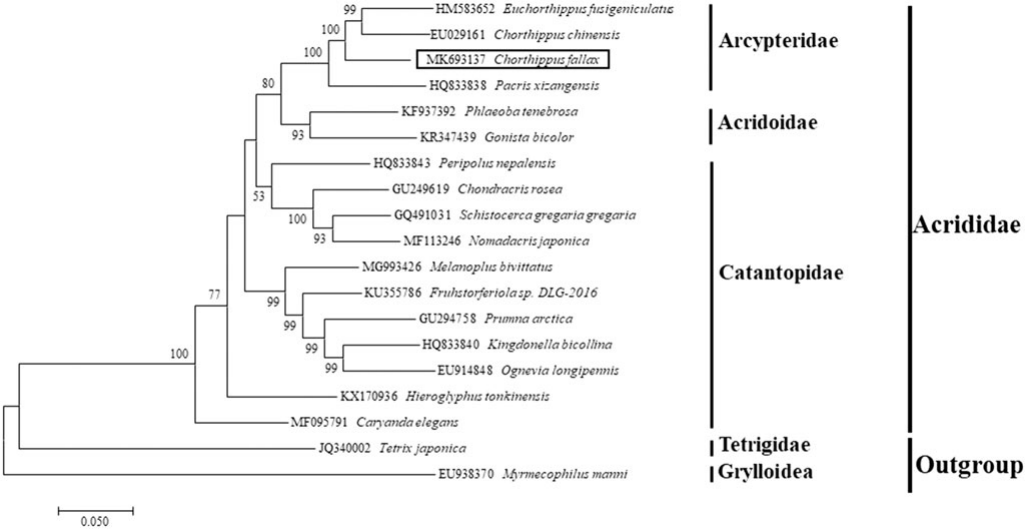
Phylogenetic analysis *of Chorthippus fallax* (Zuboxsky) based on the entire mitochondrial genome. The phylogenetic tree was constructed by using the Maximum Likelihood method based on the General Time Reversible model. Sixteen species in Acrididae superfamily and other two outgroups were selected for the phylogenetic tree analysis. The GenBank No. and species were indicated in the tree. The *C. fallax* (Zuboxsky) we identified was indicated by box.

## References

[CIT0001] Kim JS, Kim MJ, Jeong JS, Kim I. 2018. Complete mitochondrial genome of *Saturnia jonasii* (Lepidoptera: Saturniidae): genomic comparisons and phylogenetic inference among Bombycodea. Genomics. 110(5):274–282. 29191682 10.1016/j.ygeno.2017.11.004

[CIT0002] Kumar S, Stecher G, Tamura K. 2016. MEGA7: molecular evolutionary genetics analysis version 7.0 for bigger datasets. Mol Biol Evol. 33(7):1870–1874. 27004904 10.1093/molbev/msw054 PMC8210823

[CIT0003] Li R, Wang YQ, Shu XH, Meng L, Li BP. 2019. Complete mitochondrial genomes of three *Oxya* grasshoppers (Orthoptera) and their implications for phylogenetic reconstruction. Genomics. 10.1016/j.ygeno.2019.02.008 30790624

[CIT0004] Schattner P, Brooks AN, Lowe TM. 2005. The tRNAscan-SE, snoscan and snoGPS web servers for the detection of tRNAs and snoRNAs. Nucleic Acids Res. 33:686–689. 10.1093/nar/gki366 PMC1160127 15980563

[CIT0008] Sun T, Liu XL, Sun GJ, Long RJ, Liu ZY. 2016. Grasshopper plague control in the alpine rangelands of the Qilian Mountains, China. A socio-economic and biological approach. Land Degrad Dev, 27:1763–1770.

[CIT0005] Sun T, Liu ZY, Qin LP, Long RJ. 2015. Grasshopper (Orthoptera: Acridoidea) community composition in rangeland of northern slopes of Qilian Mountain (Northwestern China). J Insect Sci. 15(1):1–7. 25688084 10.1093/jisesa/ieu171 PMC4535145

[CIT0006] Wolstenholme DR. 1992. Animal mitochondrial DNA: structure and evolution. Int Rev Cytol. 141(6):173–216. 1452431 10.1016/s0074-7696(08)62066-5

[CIT0007] Zhang HL, Zhao L, Zheng ZM, Huang Y. 2013. Complete mitochondrial genome of *Gomphocerus sibiricus* (Orthoptera: Acrididae) and comparative analysis in four Gomphocerinae mitogenomes. Zool Sci. 30(3):192–204. 10.2108/zsj.30.192 23480379

